# Interpretation of psychiatric genome-wide association studies with multispecies heterogeneous functional genomic data integration

**DOI:** 10.1038/s41386-020-00795-5

**Published:** 2020-08-13

**Authors:** Timothy Reynolds, Emma C. Johnson, Spencer B. Huggett, Jason A. Bubier, Rohan H. C. Palmer, Arpana Agrawal, Erich J. Baker, Elissa J. Chesler

**Affiliations:** 1 The Jackson Laboratory, Bar Harbor, ME USA; 2 Computer Science Department, Baylor University, Waco, TX USA; 3 Department of Psychiatry, Washington University in St Louis, St Louis, MO USA; 4 Emory University, Atlanta, GA USA

**Keywords:** Addiction, Behavioural genetics, Genetic techniques

## Abstract

Genome-wide association studies and other discovery genetics methods provide a means to identify previously unknown biological mechanisms underlying behavioral disorders that may point to new therapeutic avenues, augment diagnostic tools, and yield a deeper understanding of the biology of psychiatric conditions. Recent advances in psychiatric genetics have been made possible through large-scale collaborative efforts. These studies have begun to unearth many novel genetic variants associated with psychiatric disorders and behavioral traits in human populations. Significant challenges remain in characterizing the resulting disease-associated genetic variants and prioritizing functional follow-up to make them useful for mechanistic understanding and development of therapeutics. Model organism research has generated extensive genomic data that can provide insight into the neurobiological mechanisms of variant action, but a cohesive effort must be made to establish which aspects of the biological modulation of behavioral traits are evolutionarily conserved across species. Scalable computing, new data integration strategies, and advanced analysis methods outlined in this review provide a framework to efficiently harness model organism data in support of clinically relevant psychiatric phenotypes.

## Promises and challenges in human genetics of psychiatric disorders

Psychiatric disorders are highly polygenic and show a continuous range of variation influenced by both environmental and genetic factors [1]. A major goal of psychiatric genetic research is to better understand the molecular mechanisms through which genetic variants act to influence liability to these traits. The identification of novel genetic variants provides a foothold into the complex genetic architecture that undergirds psychiatric traits. Model organisms provide an avenue into understanding the biological mechanisms that are impacted by genetic variation. In this review, we outline big data approaches that efficiently weave the vast amounts of convergent genomic data from other species into human genetic findings to elevate the likelihood of uncovering biologically meaningful pathways for further experimental follow-up and therapeutic discovery.

### The utility of genome-wide association studies (GWAS) in psychiatry

GWAS of psychiatric traits have generated an outpouring of recent discoveries in risk variant identification and polygenic prediction. From highly heritable traits, such as schizophrenia (for which >100 common loci have been reported with *N* = 150,064 [2]) to common but less heritable conditions such as problematic alcohol use (for which 29 independent loci have been reported with *N* = 435,563 [3]) and major depression (for which 102 common loci were detected with *N* = 807,553 [4]), as well as for liability across psychiatric disorders (109 loci with *N* = 727,126 [5]) progress abounds. In addition, for substance use, a recent large GWAS of tobacco smoking (*N* for smoking initiation = 1,232,091) and typical drinking (*N* for drinks/week = 941,280) has identified over 400 loci [6]. The increased power accumulated across studies of major psychiatric disorders, arising from collaborative research, has revealed clues into novel mechanisms of susceptibility to mental illnesses and substance use disorders. These large-scale GWAS have also revealed patterns of genetic variation associated with multiple disorders as well as disorder-specific loci, e.g., *CADM2* has been linked to multiple substances and common addiction mechanisms (e.g., risk-taking cognition), while the alcohol dehydrogenase genes remain alcohol-specific (e.g., [7, 8]).

### Challenges and opportunities within GWAS for psychiatric genetic studies

The recent gains in psychiatric genetic studies outlined above amplify the need to address several enduring challenges within GWAS. First, at a variant level, the bulk of GWAS “hits” fall in noncoding regions of the genome. A major advantage of GWAS as a means of discovering the biological basis of psychiatric disorders is that the lack of *a priori*, gene centric hypotheses enables discovery of trait regulatory variants in enhancer and promotor regions, lncRNAs, microRNAs, and any other molecular entity that is part of the gene-regulatory mechanism. However, in contrast to variants within coding genes, it is far more difficult to link statistically significant genetic associations to the gene products and biological mechanisms through which they act [9]. Interpretations of significant GWAS findings are complicated by patterns of related inheritance (e.g., linkage disequilibrium), such that the most strongly associated genetic variant in a locus may not be “causal” but could “tag” a true causal variant. This, coupled with long distance genomic regulation, poses challenges for unveiling specific genes and variants underlying human traits via GWAS [10]. In this review, we highlight how regulatory genetic variants can be integrated coherently with coding genes within and across species using unifying data structures.

A second challenge with GWAS is that power analyses reveal that the massive polygenicity underlying psychiatrically relevant traits and illnesses requires larger sample sizes for additional discoveries from GWAS data alone [11]. Likewise, the predictive power of a polygenic risk score (PRS), an index of aggregated genetic susceptibility to a disorder, for psychiatric disorders is also directly linked to the current statistical power of discovery GWAS [12]. However, the identification of additional trait-associated variants continues to substantially augment SNP-heritability estimates, especially in the case of rare variants, suggesting that there is more signal to be found in GWAS and sequencing studies [13], provided that higher sample sizes continue to be attained. In this review, we highlight approaches that exploit complementary data resources from model organisms that, when placed in an integrative framework with GWAS data, are showing some promise in prioritizing variants that are detected.

Third, consistent with indications from early family and twin studies, there is evidence for pleiotropy among psychiatric traits to a degree suggestive of an underlying dimension of genetic liability that parallels the general factor model of psychopathology [5, 14]. Thus, it is important to consider variants in context of both the underlying neurobiological mechanisms in which they function, and the multiple traits that are influenced by that variation to find the specific, as well as the overlapping biological mechanisms underlying behavioral traits.

A landmark contribution to our current ability to annotate GWAS signals arise from FUMA [15], a platform for functional and regulatory annotation of variants. Summary statistics from a GWAS can easily be aligned with tissue and cell-type-specific expression data and to a variety of regulatory and chromatin signatures with no computational burden on the user, making FUMA widely accessible. As an alternative to gene-based mapping techniques, software tools can also map variants to the noncoding transcriptome (e.g., LincSNP 3.0 [16]). Beyond variant mapping, harnessing multiple sources of omics data can be utilized in a multivariate framework to implicate “causal” gene sets for a disease state (e.g., SMR [17], iRIGs [18], PAINTOR [19], FOCUS [20]). Efforts are also underway, with varying degrees of success, to demonstrate to what extent similar regulatory enrichment of PRSs could enhance prediction (e.g., AnnoPred [21], LDpred-funct [22]). However, most of these approaches have been limited to human genetics and genomics data. In this review, we highlight approaches that bring together the breadth and depth of well-controlled model organism studies that place genetic and genomic findings in biobehavioral context that can expand on this or other interpretive tool sets.

## Multi-species genomics to address challenges in GWAS variant interpretation

Across these historical and contemporary research challenges, Big Data approaches that harness information from additional sources, including cross-species genomic analyses, can provide elegant solutions to current barriers in psychiatric genetics [18, 23]. It cannot be understated that we need better-powered GWAS, especially as we look to polygenic scores as a means of leveraging the modest effect sizes from GWAS. However, increasing the sample size alone may be merely a theoretical solution for certain traits where rare variation and modest effect sizes contribute substantially. Incorporating evidence from molecular and cellular biology shifts the focus of genome-wide analyses from variant detection and identification to evaluating the relative contribution of a prioritized subset of loci. This helps control the family-wise error rate, thus increasing power, and provides context about the genome at multiple levels (i.e., structure, function, and regulation) while also accounting for the polygenicity of a trait. Leveraging information from annotated genomic regions that affect gene function was shown to robustly increase the power to identify genomic associations across 27 human traits [24].

There is extensive information available from human and model organism functional genomics that may be brought to bear on human GWAS findings in the context of specific behaviors, tissues, and molecular mechanisms [25, 26]. Prior to the widespread availability of human ‘omics data, some of the earliest efforts to characterize the mechanism of variants detected in human association studies relied on expression of orthologous genes from studies performed in animal models. The rich data resources from these studies continue to be valuable due to the breadth and depth of studies that are possible in animal models, under precisely controlled conditions of drug exposure and other neurobiological or behavioral processes. Further, model organism data also contain a rich source of expression regulatory information including expression quantitative trait locus (eQTL) and epigenetic data from many tissues and brain regions, some of which is collected in populations that facilitate the global correlation of transcript abundance to neurobiological and behavioral parameters [27]. Integration of functional genomic information from multiple species into GWAS provides new clues about the biological context and consequences of genetic associations and PRSs, and provides insight into how to model such variation in in vivo preclinical models with intact central nervous systems and expression regulatory machinery.

Below, we illustrate the promise of harnessing these model organism data, for which decades of comparative behavioral research has produced numerous experimental paradigms aimed at consilience, such as drug self-administration and response studies across multiple mouse and rat populations in genetics and genomics [28]. We propose methods for integrating valuable and ever-expanding complementary model organism and human genetics and genomics data (such as GTEx [29] and GeneNetwork.org [30], psychENCODE [31] and modENCODE [32]) and highlight new approaches for boosting power in human genetics through Bayesian inference in heritability and polygenic analyses, outline exciting developments aimed at bridging the “analytic currency” gap between human and model organism research, and present some technical and philosophical challenges. The overarching goal of this review is to focus on ways in which we might utilize the complementary strengths of human and animal genetics to advance their common research mission: gaining a better understanding of the biology of complex traits.

### Potential and challenges for model organism data integration

There is considerable and growing interest in employing nonhuman animals to meet some of the challenges for human genetics outlined above. There is a tremendous depth and breadth of model organism genetics and genomics studies spanning many areas of behavioral and neurobiological parameters. These include differential expression studies following various behavioral and drug exposure paradigms [33], large-scale screens of gene-targeted deletion mutants [34], and genetic studies in populations such as the BXD RI mouse lines [35] and inbred strain panels [36], which often combine gene expression and genetic analysis. Numerous QTL positional candidates have been identified from a large number of behavioral and neurobiological mapping studies [37]. Selective breeding in rats and mice have been able to separate alcohol preferences [38, 39] and chronic use/withdrawal [40]. These data provide a rich backdrop and context in which to interpret the more global phenotype or disease information that is the frequent subject of GWAS analysis.

Animal geneticists have a rich history of using model organisms to study behavioral traits that mirror aspects of human psychopathology. Many of the genes and variants identified in model organisms are also now also being found in human GWAS studies (Table 1), indicating that convergence of these studies is feasible. To date, model organism evidence has largely been used as a form of post-GWAS validation to characterize significant SNP/gene effects (e.g., [41, 42]). There have been a few promising recent examples of model organism research that, when coupled with human GWAS findings, have revealed insights into the biological mechanisms underlying psychiatric disorders. Model organism data have also produced experimental insight into disease mechanism. For example, researchers used mouse models to study the effect of a particular protein, complement component 4 (C4), on synaptic mediation during development [43]. By using a mouse model in conjunction with convergent evidence from human genomic studies, researchers were able to study the effects of *C4* gene deficiencies on synapse elimination during postnatal development in a way that is not possible in humans. Researchers are beginning to leverage model organism genomics directly in the context of human genetic studies. For instance, gene co-expression networks associated with mouse neurodegeneration phenotypes demonstrated enrichment for human GWAS associations with Alzheimer’s disease [44]. Integrative methods for jointly analyzing model organism data directly with human GWAS are under active development. One recent example identified novel brain mechanisms of alcohol use and dependence by coanalyzing human GWAS, human protein–protein interaction networks, and mouse gene co-expression data. In doing so, the researchers interrogated ethanol-responsiveness genes obtained from mouse gene expression data of the PFC, VTA, and NAc [45].

**Table. 1:** Genes identified in both human GWAS and model organism genetic studies.

Human gene	Model organism	Year of publication	Model organism publication (PMID)	Trait	Date	Human publication (PMID)	Trait
MPDZ	Mouse	2002	11978849	Alcohol withdrawal	2009	19175764	Alcoholism
MC1R	Mouse	2003	12663858	Analgesia	2003	12663858	Analgesia
OPRM1	Mouse/Rat	1994/1998	7982048/9512064	Pain genetic variation/alcohol intake	1998	9756053/9689128	Alcohol dependence/opioid binding, addiction
GAD1	Mouse	1994	8974318	Alcohol withdrawal	2007	17034009	Alcoholism
CHRM5	Mouse	2002	11900778	Increased drinking	2004	15292665	Schizophrenia
GABRB2	Mouse	2003	12490572	Action of alcohol	1999	10195814	Alcohol dependence
ALDH2	Rat	1991	2053491	Alcohol drinking behavior	1982	7180842	Alcohol metabolism Caucasian/Asian
ALDH1	Mouse	1996	4015840	Alcohol metabolism inbred	1983	6354999	Alcohol metabolism Caucasian/Asian
FAM53b	Mouse	2016	26581503	Cocaine	2014	23958962	Cocaine dependence
PPP1R1B	Mouse	1998	9694658	Drugs of abuse	2006	16237383	Amphetamine experience
CSNK1e	Mouse	1999/2005	10591541/16104378	Amphetamine/cocaine-induced stimulation	2006	16237383	Amphetamine experience
COMT	Mouse	1975/1998	1185192/9707588	Differential seizure susceptibility/KO social behavior	2003	12716966	Methamphetamine brain response variation
DBH	Mouse	1991/1999/ 2000	1684202/10594079/ 10777779	Altered norepinephrine and serotonin/seizure/alcohol	2000	10673769	Cocaine-induced paranoia
DRD1	Mouse	1994	8001143	Cocaine behavior	1997	9154217	Addictive behavior
DRD4	Mouse	1997	9323127	Supersensitive cocaine	1993	8216280/8268330	Alcoholism/delusional behavior
DBH	Mouse	1992/2000	1542654 /11093800	Ethanol induced	2000	10975602	Smoking cessation
DDC	Mouse/Fly	1986/2006	3703899 /16783013	Drug studies locomotor behavior	2005	15879433	Nicotine dependence
HTR3A	Mouse	2001	11685380	Conditioned place preference	2001	11207027	Schizophrenia and bipolar
HTR5A	Mouse	1999	10197537	Activity/lsd	2009	19328558	Bipolar
ARRB2	Mouse	1999	10617462	Morphine analgesia	2006	16894395	ADHD
GRIN3A	Mouse	2005	15866554	PPI	2009/ 2011	20016182/ 20084518	Alzheimer/nicotine
NRXN1	Mouse	2009	19822762	PPI, learning, grooming	2005	16451640	COGA
HP1BP3	Mouse	2016	27460150	Cognitive aging			
DAT1	Mouse	1998	10195128	Cocaine IVSA	2001	11449401	ADHD
GRIN2B	Mouse	1996	8789948	Abnormal startle	2000	10945659	ADHD, ODD and conduct disorder.
CHRNA3	Mouse	1999	10318955	Megacystis-microcolon-intestinal hypoperistalsis	1998	9758605	Epilepsy
CHRNB4	Mouse	2004	14996991	Seizure	1998	9758605	Epilepsy
CHRNA6	Mouse	2002	11927835	Nicotine	2002	12195439	Epilepsy
CHRM1	Mouse	2001	11752469	Hyperactivity	2003	14504414	Psychiatric symptomology
CHRM2	Mouse	1999	9990086	Impaired drug response	2002	12116189	Depression
CYP2A6	Mouse	1989	2733794	Altered metabolism	1998	9655391	Nicotine metabolism
CYP2B6	Mouse	2010	19923441	Nicotine pharmacokinetics	1992	1736885	Drug metabolism
NTRK2	Mouse	1993	8402890	Neonatal death	2005	15838534	Eating disorder
SHC3	Mouse	2005	15716419	Spatial memory	2007	17179996	Nicotine
DNM1	Mouse	2007	17463283	Abnormal motor capabilities/coordination/ movement	2008	18806795	Exercise-induced collapse
TAS2R38	Mouse	2014	mousephenotypes.org	Limb grasping	2005	15466815	Taste
APBB1	Mouse	2004	14689444	Abnormal spatial learning	1998	10079843	Alzheimer disease
NRG3	Mouse	2016	27606322	Abnormal behavior	2008	18708184	Schizophrenia
DRD2	Mouse	1995	7566118	Impaired coordination	1991	1832466	Neuropsychiatric disorders

Despite this substantial progress, there remain conceptual and technical challenges for data integration across species. These occur at the levels of phenotypic comparison, genetic conservation, and computational scale. A major challenge at the phenomic level is that any effort to integrate evidence across model organisms and humans must acknowledge that human psychiatric diagnoses and classifications are often based upon clinical instruments and nosology that are not easily transferable to model organisms, therefore efforts to “diagnose” animal models are discouraged. However, it is apparent that aspects of a disorder can transfer across species and be easily captured with experimental data, and increasingly, GWAS of psychiatric disorders are providing corroborating support for variants that influence both disorders and their trait-like manifestations that may be recapitulated in model organisms [46]. For example, it was recently shown that ethanol responsive genes in mouse prefrontal cortex, nucleus accumbens and ventral tegmental area were overrepresented in GWAS for alcohol dependence in the Irish Affected Sib-Pair Study of Alcohol Dependence and the Avon Longitudinal Study of Parents and Children [26]. The identification of network-level associations between humans and mice suggests shared sensitivity in ethanol responding, and thus can serve as support for nominal GWAS signals. However, far more complexity and heterogeneity than ethanol response underlies alcohol dependence in humans. Recent genomic distinctions identified between the consumption (AUDIT-C items 1–3) and the problematic (AUDIT-P items 7–10) subscales of the Alcohol Use Disorder Inventory Test (AUDIT) [8], [47] echo similar findings in model systems, the data from which will be critical for the interpretation of molecular mechanisms [48].

There is concern that comparative, multispecies approaches will not be as readily feasible for certain psychiatric traits. Behavioral characteristics including speech, language, and certain executive and metacognitive functions are also impossible to assess in model organisms. It should be noted that, most studies that attempt comparative genomics across species are based on limited genetic diversity, often comparing a single idiosyncratic strain to a small sample of the population of humans, e.g., [49], and therefore cannot discern between individual differences within populations and between species. For some disorders, there is a substantial role of brain structures that are under developmental control of poorly conserved genomic regions, leading to significant cross-species differences in these structures [50]. This potentially could preclude detection of genetic variants that regulate disorders through effects on the development of these structures. Following this logic, some aspects of substance use disorders are served by neural structures that show more conservation and may be more likely to provide convergent mechanistic evidence for overt characteristics of drug intake, withdrawal, compulsive responding even with choice and punishment, but perhaps not “desire to quit” or other metacognitive and psychosocial aspects of addiction.

However, all psychiatric disorders including SUDs are highly complex traits likely involving many risk loci. Some of these effects are manifest across species, even if the end result in humans includes behavioral output not readily observable in nonhuman model organisms. Therefore, one can model the effects of genetic risk variants on more proximal biological consequences; for example, one might study the influence of C4 variation [43] on endophenotypes captured in Research Domain Criteria (RDoCs) including synaptic excitability, or neuronal reactivity and the various startle phenotypes it is associated with, but not all of the species-specific cognitive and behavioral output that are central to the disease pathology. Historically, the field has been distracted by pharmacologically predictive characteristics that have little face validity with the disorders to which they are applied [51]. Below we describe how cross-species comparative genomics provides a tool that can be used to identify what aspects of the human disorder are reflected in model organism genomics, allowing data-driven discovery of the relations among traits across species [52].

At the genetic conservation level, cross-species genetic research has been hindered by the “analytic currency” problem. Human geneticists typically work at the variant level, and genomics data, particularly from expression studies, are often reported at the gene or transcript level. Prior efforts at model organism follow-up of human GWAS data were limited to human variants that could be positionally assigned to a gene, but as described below, this is no longer the case. As is evident from regulatory mapping analyses, the action of a variant does not readily correspond to the most proximal gene, or even a single gene. Further compounding the problem, noncoding regulatory variants are often found in poorly conserved regions of the genome, which renders cross-species gene orthology mapping challenging and variant mapping through sequence alone, impossible in many cases. Therefore, approaches that exploit both gene orthology and convergence of variant regulatory relations are most promising toward relating trait regulatory variation across species.

In the case of intragenic variants, current methods use transcript and protein annotations to identify causal SNPs based on the severity of mRNA and protein modifications [53] and other functional consequences [54]. However, the majority of SNPs are intergenic, suggesting the involvement of distal gene-regulatory mechanisms (e.g., chromatin accessibility). Therefore, the common approach of associating SNPs to nearby downstream and upstream genes can elicit false positives [55] and therefore it is necessary to use data from gene eQTLs, epigenetics, and 3D genomics to assess the relationships among regulatory variants and their distal targets. Although most prior variant-to-gene annotation efforts have relied on positional approaches, i.e., assigning SNPs to genes based solely on physical proximity (e.g., MAGMA software [56]), modern approaches in humans rely on extensively curated functional and regulatory mapping from ‘omics data (e.g., S-PrediXcan software [57], TWAS [58], Hi-C coupled MAGMA or H-MAGMA software [59]).

**Fig. 1:**
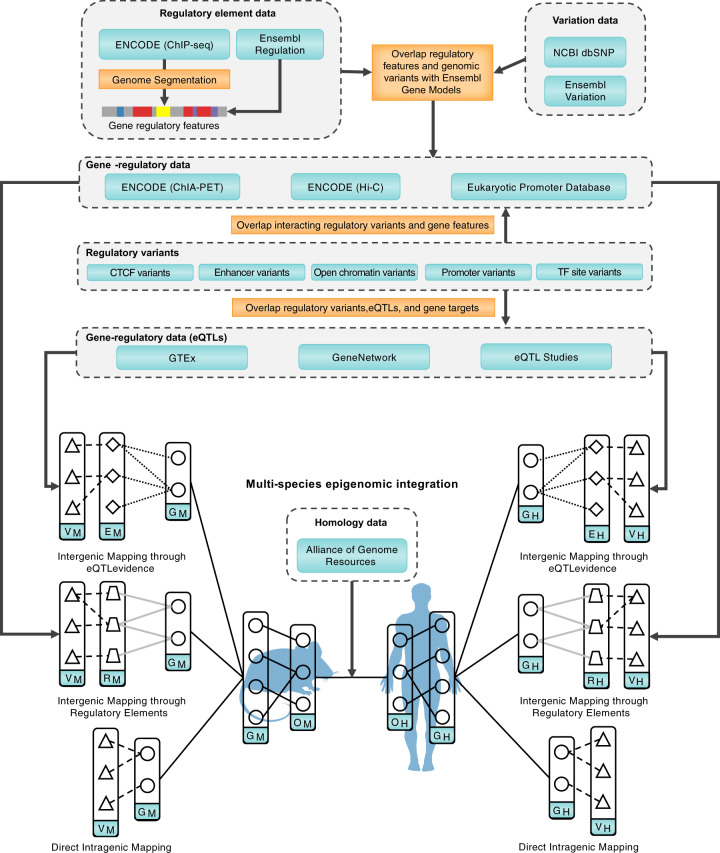
Multispecies genomic and epigenomic data integration. Genetic variation, gene regulation, and homology datasets are retrieved from a variety of publicly available resources and data repositories. Human (V_H_) and mouse (V_M_) variants are connected to the gene (G_M_, G_H_) that either contains a coding variant or is regulated by a noncoding variant. Epigenetic markers and regulatory features (R_M_, R_H_) are retrieved from ENCODE and Ensembl, then overlapped with genetic variation data from Ensembl and NCBI in order to identify regulatory variants (V_M_, V_H_). Regulatory variants (V_M_, V_H_) are overlapped with gene-regulatory datasets in the form of eQTLs (E_M_, E_H_; processed from GTEx, GeneNetwork, and specific mouse populations) and chromatin interaction studies (e.g., ChIA-PET experiments from ENCODE and gene-promoter interactions from the Eukaryotic Promoter Database). Association of regulatory variants and gene-regulatory information allows for the identification of putative gene targets. These datasets are harmonized within-species for mice (V_M_, E_M_, G_M_, R_M_) and humans (V_H_, E_H_, G_H_), then related across species through orthologous gene targets (O_M_, O_H_) derived from homology resources like the Alliance for Genome Resources.

However, all of these approaches have almost exclusively used data from human genomic analyses. Similar approaches have been deployed in model organisms, but the integration of resources across species has remained rather incomplete, limiting the approach to a small number of applications. To facilitate cross-species analysis, integrative data analyses have historically relied on gene homology associations from model organism databases [60] and gene orthology services [61]. Analysis involving multiple species therefore most often occurs at the gene level, introducing a GWAS-specific integration challenge: the need to associate genetic variants with genes. For complex disorders, such as schizophrenia and SUDs, this often requires characterization of the regulatory nature of genetic variants associated with disease, or identifying functional variants in submolecular domains of drug targets that could confer vulnerability or resistance to various treatments. However, noncoding regions of the genome are often very poorly conserved across species, and the targets of the variants can be far away. Moreover, many of the implicated noncoding variants in GWAS reside in gene expression regulatory regions [62]. Here, we highlight solutions for the assessment of conserved effects of variants through their orthologous genomic targets to support a wide-range of applications in integrative functional genomics (Fig. 1).

## Solutions for data-driven cross-species analysis

Broadly speaking, integration of multispecies functional genomic data can occur in two ways—from the phenomic or genomic orientation. For example, top-down, trait-based approaches to cross-species analysis utilize the similarity of human disease-related phenotypic profiles to model organism phenotypic profiles to identify gene-disease associations [63]. These approaches, embodied in resources developed by The Monarch Initiative [64] identify similar phenotypes across species through integrated ontologies and semantic similarity methodologies that apply semantic reasoners to a unified knowledge graph [65]. Such phenotype-driven approaches, which leverage multispecies data, have been effective at assisting rare disease diagnosis [66] and improving identification of causal genetic mechanisms [67], but these approaches are challenging to apply in the context of high phenotypic and genetic heterogeneity due to the extensive differences among species in the behavioral manifestations of neurobiological variation.

In highly complex psychiatric disorders in which model organism traits may only capture a facet of the human disease, alternative bottom-up strategies that aggregate genomic data may be more suitable for identification of the driving genetic mechanisms associated with complex traits and disease. The varieties of biological entities—genes, proteins, variants, methylation sites, and chromatin states for example, which can be characterized via genome-wide experimentation, pose a challenge for integration and analytic efforts [68]. These challenges may be mitigated via combinatorial integration of fundamental data attributes into generalized data structures that can be mined for patterns or emergent gene-disease relationships. GeneWeaver [69], for example, relies on a bipartite data model [70] and heterogeneous data networks [71] to integrate and analyze functional genomics data such as differential expression studies, GWAS, curated annotations, and QTL mapping studies through a single data structure that facilitates aggregation of information. Harmonizome [72], on the other hand, aggregates functional genomics studies from a variety of sources by implementing an association matrix across shared attributes and relying on machine learning approaches to identify novel patterns.

Fundamental integration through knowledge graphs may also be applied to large-scale heterogenous analysis. KnowEng [73] uses a knowledge network to navigate the integration of statistical experimental data and contextualized user information to identify human and mouse interactions. Aggregated knowledge networks can be analyzed using traditional network mining approaches or machine learning. Other tools, such as HumanBase [74] or the DIAMOnD [75] algorithm, also take advantage of traversing large ad hoc networks of functional connectivity. Networks are navigated through machine learning or association matrices to connect multispecies gene or variant relationships.

There are many approaches to cross-species comparative genomics and phenomics integration (e.g., Table 2) and analysis must optimize among competing needs of computing scalability, data accessibility, and data scope. For example, the sheer number of variants in humans and rodents and the unbounded phenotype dimension lead to the problem of phenomenal computational scale. The tremendous heterogeneity of model organism datasets, from mutation characterization studies, curated pathway and gene annotation sets, and extensive genetic and genomic data at the level of genes and variants, presents a problem of size, scope, and complexity, in the realm of big data problems, requiring computationally scalable solutions.

**Table. 2:** Tools for Functional Genomics Data Integration.

Tool Name	Description	Strategy
AnnoPred	Estimates PRS using genome-wide variants that are differentially weighted based on the integration of evidence across GWAS summary statistics and multiple annotation resources for different tissue types, genomic features, and the functional assessment of SNPs.	Bayesian framework integration
DIAMOnD	This tool identifies potential variant-to-gene associations based on module inclusion. Uses an algorithm for detecting disease modules based on network connectivity.	Algorithm for network module analysis
ENCODE Screen	Useful for discovering the potential regulatory role of genetic variants using *cis*-regulatory elements from ENCODE data in human and mouse.	Database
FOCUS	Used to determine gene–trait associations from transcriptome-wide annotation studies using LD among SNPs and eQTL weights embedded in a probabilistic model.	Probabilistic systems framework
FUMA	Online tool to visualize and aggregate positional, eQTL, and chromatin interaction maps to perform enrichment analysis of human GWAS data. Can be used to associate genetic variants to target genes based on eQTL and chromatin interaction studies.	Tools pipeline and visualization
GeneNetwork	Set of variant, expression, and eQTL multispecies tissue specific datasets used to link genetic maps to disease and phenotypes of interest.	Database, statistical and probabilistic tools
GeneWeaver	Multispecies data integration tools that allow users to identify putative genes of interest based on shared or unique genetic or variant data of interest. Tools available to map, manage, and analyze large datasets.	Bipartite, k-partite, combinatorics, network analysis
H-MAGMA	A modified version of MAGMA that extends gene-to-variant mapping by including long-range loci interactions predicted by Hi-C.	Statistical multiple regression models
Harmonizome	Online resource for data integration from existing genomic resources.	Association matrix, machine learning
HumanBase	Online tools for tissue specific gene and network interactions.	Association network, machine learning.
KnowEng	Integrative analysis following formatted pipelines for knowledge discovery.	Knowledge network, machine learning
LDPred-funct	Used to derive polygenic scores using multiple genetic variants. LDpred-funct estimates polygenic effects by employing a model that accounts for LD and identify trait-specific priors that are based on posterior casual associations.	Probabilistic modeling
MAGMA	Software tool used to assign GWAS identified variants to genes, based on physical proximity, and perform joint and conditional association models that examine gene-, gene-set, and interaction effects.	Statistical multiple regression models
modENCODE	Collaborative data set for genomic functional elements across several species, used to define genomic regions and variants of interest.	Database, ModMine toolset
Monarch	Semantic integration of phenotypic disease associations to identify underlying genes.	Knowledge graph
PAINTOR	Used to determine SNPs to be tested for phenotypes of interest. Predicts the impact of multiple casual variants on genomic annotations by incorporating summary associations statistics, functional annotations, and LD statistics.	Probabilistic systems framework
psychENCODE	Collaborative data set for genomic functional elements, used to define genomic regions and variants of interest in the brain.	Database, ModMine toolset
S-PrediXcan	Used to predict gene associations to disease using gene expression levels to mediate summary GWAS and measured transcriptome studies without the need to use individual-level data.	
SMR	Identifies genes with expression levels and pleiotropic associations with diseases of interest via the integration of GWAS variants and expression data derived from eQTL studies.	Mendelian randomized analysis
TWAS	Identifies expression–trait associations by creating putative transcriptome-wide associations derived by integrating gene expression measurement with GWAS estimated associations.	

## Big data and the integration of human and model organism studies in psychiatric genetics

Cross-species analysis typically happens at the level of abstracted relations among variants or genes and can thus be quite reduced in scale. However, (1) the scope of genomic studies is completely unbounded and it is possible to find hundreds, if not thousands of animal studies of disease-relevant neurobiology and (2) the parsing and representation of genomic variants from diverse data sources and their mappings onto one another does not scale so easily. Retaining this traceable mapping while allowing integrative and interactive analysis is a problem of high complexity and scale. The storage, analysis, distribution, and integration of human and model organism functional genomic data are especially challenging, as they embody typical problems encountered in the big data world [76] often referred to as the four V’s of data—volume, variety, velocity, and veracity.

The sheer volume of data required to support comprehensive cross-species data integration of genes and individual variants is staggering. For example, if we assume that the average number of coding genes in mammalian genomes is ~25,000, then constructing rudimentary connections among the genes in five species would produce 1/2*n*(*n*−1) relationships, where *n* is the number of genes in the network. If represented as a graph, with each edge representing a relationship, the graph would be enormous but tractable, comprising ~7.8E9 edges. But, the genome is only one dimension of the problem. The other is the sheer number of contexts in which that genome is experimentally profiled. With thousands of human and model organism addiction genomics datasets, and hundreds of thousands of species-specific pathway data, brain regional transcriptomes and other relevant data resources, one quickly reaches a problem requiring scalable solutions. Analysis of a handful of organisms can therefore be handled with large, conventional high-performance computing systems. At the variant level, however, the relationship problem is greatly compounded. Known variants, which outnumber genes within the typical model organisms by more than 20,000–1, would naively require ~1.25E17 edge relationships. While intelligent approaches for computing on large graphs, such as taking advantage of partitioning [77], sparse connectivity [78], or heuristics [79], can aid in the management and analysis of these relationships, exhaustive examination of static graphs of this potential size is intractable due to computing limitations, storage, and real-time accessibility. As the number of genomic experiments continues to grow, particularly in the model organism space, one viable option may be the dynamic analysis of datasets using elastic on-demand cloud services that make use of horizontally scalable computing to efficiently distribute computing tasks to address very specific questions.

A corollary to the volume/variety of data associated with variant mapping across species is the velocity at which it is produced, and, subsequently, the rate at which it must be collated, curated, and made accessible. With over 4500 eukaryotic genomes assembled over the last decade [80], it has been argued that genome-scale data will be bigger than Big Data associated with astronomy, YouTube, and Twitter by 2025 [76]. To complicate the processes used to integrate the vast scope of data are data sharing policies that historically do not require automated sharing of model organism data, resulting in data analysis processes that result primarily from ad hoc relationships [81]. To mitigate the stresses imposed by data velocity, it is critical to devise a means to access, integrate, and dynamically update these data in a manner that avoids redundancies and keeps data provenance intact. While it is inevitable that there will be an uneven integration of data from a variety of sources, it is incumbent on the bioinformatics community to create systems to rapidly track intentional methodologies for data cleaning and reduction through the discovery of duplicated or deprecated data.

**Fig. 2:**
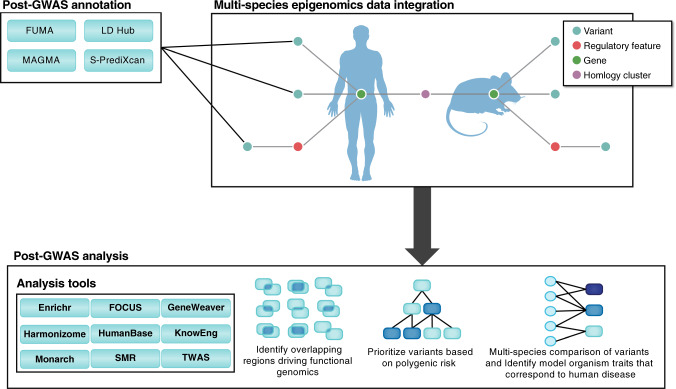
Multispecies genomic and epigenomic analysis. Species-specific gene, gene-regulatory, and variant-level data are harmonized from public resources. Using variant and gene annotations as input from post-GWAS annotation tools (e.g., FUMA, MAGMA, etc.), gene-regulatory components can be related across species via epigenomic modeling. Gene targets identified from epigenomic modeling can be used for further post-GWAS analysis with tools such as Enrichr, GeneWeaver, KnowEng, etc. Such analyses have numerous biomedical applications, such as the discovery of disease-relevant model organisms and traits.

By addressing these problems in Big Data, scalable applications in integrative functional genomics for psychiatric genomics are enabled (Fig. 2). The integrated, global mapping of trait regulatory variants across species through target genes can facilitate the integration of model organism genomic data to fill the mechanistic knowledge gap between noncoding human genetic variant and human disease. This integration can be accomplished through the aggregation of curated and high-throughput experimental data from multiple domain-specific resources. Data resources such as GTEx [29], ENCODE [82], and Roadmap Epigenomics [83] provide extensive coverage of genomic regulatory features and gene-regulatory mechanisms. High-level regulatory features including CTCF binding sites, enhancers, open chromatin, promoter, promoter flanking, and transcription factor binding site attributes can all be retrieved from regulation databases [84]. These features can be annotated to genomic variants from the Ensembl variation database [85], for example, to identify regulatory variants within regions of interest. Identifying putative regulatory interactions between regulatory variants and genes can be accomplished through layering several approaches. Topologically associated domains, verified from Hi-C studies and integrated from published studies and the ENCODE resource, can be used to delineate putative gene-regulatory boundaries and all combinations of regulatory variants and genes that are associated within the boundary. Experimentally confirmed feature-gene interactions mediated by RNA polymerase II and identified using ChIA-PET studies, sourced from ENCODE and various publications, can also be used. Finally, eQTLs can identify variant influences on specific genes.

Compounding the issues encountered by the complexity of raw data is the potential for underlying data bias and the subsequent difficulty of attributing veracity to the data. There is an implicit bias in the sampling of genes represented in an experimentally derived genomic data set because each genomic technology and especially a curated genomic data resource is based on a different breadth, e.g., individual mutation studies curated from literature by the model organism databases vs. genome-wide gene expression by RNA-seq data. Differing approaches affect the rate of false positives in the data set. For example, QTL positional candidate sets may have many genes with likely only one or a few true positives, in contrast to differential gene expression sets for which the statistical threshold defines a false discovery rate. Semiquantitative or quantitative scores for these datasets need to be created to reduce our reliance on qualitative scoring. Enrichment analyses and systems genetic correlation tools suffer from annotation bias in that one often retrieves results representing areas of investigation that are dense with information, resulting in apparent patterns and trends that are an artifact of coverage. Data resources like GTEx also suffer from biases based on uneven sample size, and the particular tissues and conditions investigated. The net effect of the uneven statistical power in these data resources is to upwardly bias well-powered but less relevant findings, in which tissues or phenotypes are spuriously associated with disease. Therefore, it is important to consider error-rate controls, and other procedures, but also the uniformity of analysis in the data used in analysis.

Multitissue eQTL data can be integrated to provide context-specific variant mapping. Primarily derived from the GTEx project or model organism resources such as GeneNetwork [30], data from mouse, rat, and human genetics experiments represent a diverse and deep pool of data. Single cell RNA (scRNA) enables the exciting possibility to investigate eQTLs and gene co-expression in complex, multicellular tissues. For example, scRNA sequences have been used to create high fidelity classifications of brain regions based on local variants [86].

Furthermore, scRNA has been used to identify cell-type-specific *cis*-eQTLs and variant co-expression networks [87]. Gene expression genetics studies in model organisms have tremendous precision with new populations like the Diversity Outbred segregating 45 million mouse genetic variants comprising 90% of the known mouse genetic variome [88]. Recombinations are at extremely high precision, and large mapping population sample sizes for an increasing number of brain regions, and the derivation of this population from eight founder strains provides a means of reducing eQTLs to a small handful of regulatory variants at the SNP level [89]. As such, it is possible to identify eQTL variants that may affect one of several gene-regulatory mechanisms targeting a human orthologue, and to assess its effect on mouse phenomics, cellular gene expression, or other endpoints in silico, in vitro, or in vivo. Many of these tools provide browser-based and limited scriptable interfaces with continued adoption of new technologies, but exposing model organism eQTL data to large-scale dynamic tools for graphical integration would be of tremendous utility in readily enabling facile interrogation of variant-gene relations.

## Multiple applications are readily possible with integrated data structures

A compelling approach to the prioritization of GWAS variants enabled by Big Data integration is the use integrated cross-species data to identify and characterize those variants with a known mechanistic role in neurobiological pathways to disease, or to identify human variants with highly specific hypothesized roles in particular cases of disease, such as the widely studied ADH1B in AUDs. Although current applications and analytic implementations do not fully take advantage of large-scale data resources, the emerging scale of data and high-volume comparative analyses will most certainly merit scalable approaches in the near future. Most present approaches do not yet harness the full capacity of cross-species comparative analyses at scale, and initial applications have been necessarily focused on small, single locus problems. However, these simple applications are ripe for extrapolation to global questions about the neurobiological mechanisms of addiction. One promising application of multispecies epigenomic integration is comparative gene regulation. Now that characterization of gene-regulatory components (e.g., enhancers, TF binding sites) and their putative gene targets is improving, integrative methods can identify shared genomic regulators across species. In one example, from studies on alcohol dependence and cholesterol, at least 4000 SNPs from human GWAS can be mapped onto the mouse genome [90]. Furthermore, some of these SNPs, which are involved in human liver function, can be mapped to liver-specific enhancers in mice [91]. This type of comparative analysis could be used to identify convergent regulatory features and variants across species, enabling the development of mouse models for testing SNP causality in humans. Integrative systems have successfully been used to identify disease-relevant genes and to identify gene-regulatory SNPs involved in alcohol preference and withdrawal involved in epigenetic regulation in mice at a distal enhancer element [92]. Query of public genetic data resources indicates that variation in the same gene occurs in humans, via a promoter variant, rather than an enhancer [93].

Several recent approaches have been developed for prioritization of disease-relevant genes and variants from integrative omics analysis. These tools utilize large integration pipelines coupled to networking and statistical tools to establish a relative importance (e.g., priority indexing) of variants across tissues of interest focusing on immune-mediated traits. For example, Wang et al., develop a risk gene selection method, called iRIGs [18], which incorporates GWAS and a number of genomic features including expression, chromatin interactions, and gene-regulatory data into a Bayesian framework for prioritization. This framework prioritizes genes within a small 2 Mb region near risk loci identified from GWAS using a select set of epigenetics including promoter, enhancer, and chromatin interactions from Hi-C studies. A similar approach, developed by Fang et al., utilizes a priority index (Pi) pipeline [94] designed to prioritize genes from GWAS variants for specific immune traits. Pi combines genomic predictors in the form of gene proximities, chromatin interactions, and expression modulation evidence (eQTLs) with network-based models to prioritize trait–gene associations. To date, these approaches have not been applied to model organism data, but they most certainly can be. Furthermore, with the implementation of cross-species variant mapping such as those presented in Fig. 1, they can exploit the broad, heterogeneous multispecies data corpus.

Another application is to compare sets of trait-associated human and model organism genomic data to identify similarly regulated disease-relevant traits suitable for convergent validation experiments. Mapping of human disease-related characteristics onto model organism behaviors has been a controversial area of research, and for many, the perceived relevance of animal models is hindered by ever-refined definitions of face validity [95]. This argument misses the point that a model is by definition a simplification of a system that renders it amenable to particular types of study, including validation. Animal models, themselves, have successfully been used to measure the efficacy of drugs and validate various drug targets [51]. Further, there may be sufficient consilience between human disease traits (such as the various aspects of alcohol use disorders) and those modeled in animals (e.g., ethanol intake) at a genomic level (rg = 0.77 between problem drinking and typical alcohol intake [3]) to allow for careful cross-species data integration for these subfacets of human disease. Research targeting behavioral mechanisms that do converge across species does not discount or diminish the need to study the remaining complexity in the human phenotype. Rather, it serves as a powerful means of discovery of the nature of vulnerability and resilience to those components of psychiatric disorders that, in their many manifestations and potentially relevant classifications, are amenable to biological insights, and thus, promising targets for therapeutic discovery.

Finally, the prioritization of variants for use in polygenic risk analysis can be refined. Savvy integrative methods can be combined to achieve sets of variants that meaningfully contribute to trait variation from a broad network of genes. Aggregating across the tools and databases listed in the current review will help researchers to match (1) variants to genes, (2) genes to biological functions, (3) functions to plausible molecular mechanisms—ultimately achieving more robust effects with high signal-to-noise ratios—and (4) traits and disease characteristics within and across species. A few studies [96] have constructed polygenic scores from variants in genes known for disease pathology or targets co-expressed with putative trait genes from relevant brain tissues (via GeneNetwork), both of which demonstrated increased prediction than a random sets of genes and achieved trait specificity in mice [96] and humans [97]. But not all biologically informed polygenic scores exhibit significant prediction [98] and these methods have not been benchmarked with classical approaches selecting specific statistical criterion (e.g., *p* value threshold; PRSice [99]) nor approaches that combine both statistical and alternative biological information (e.g., LD; LDpred [100], PleioPRED [101], AnnoPred [21]). A mixture of these techniques is likely required to best inform gene and variant prioritization in human GWAS studies.

## Future research directions

The multiple strategies we have outlined can be used to address the challenges and opportunities for the integration of diverse model organism datasets to augment the interpretation of GWAS and define genes and molecular pathways that underlie aspects of psychiatrically relevant phenotypes. Heterogeneous functional genomics leverages the combined information in population genetic diversity, systems biology, gene-regulatory analysis, and advanced phenotypic measurements to identify and characterize mechanisms of psychiatric disorders of the greatest complexity. Much work remains to facilitate dynamic data integration across these data types. The continued generation of adequately powered and broadly unbiased data resources in neurogenomics is essential across multiple species. Data sharing policies and practices along with platforms for data sharing and data integration are required. Community standards and practices that make data findable, accessible, interoperable, and reproducible need to be adopted and resourced so that all researchers engaged in the generation and analysis of integrative functional genomics data have the capability of contributing to and benefiting from data integration. Development of analytic approaches and algorithms are also required for diverse applications in functional genomic data integration. Scalable computational solutions that allow for such high dimensional data integration will enable a growing array of tools and approaches for the discovery of unknown mechanisms underlying psychiatric disorders, providing a more complete understanding of disease mechanisms.

## Funding and disclosure

AA and ECJ received support from NIH MH109532; DA32573 (AA); F32AA027435 (ECJ). EJC, TR, EJB, and JAB received support from NIH AA018776; and EJC from P50 DA039841. RHCP, JAB, and SH received support from NIH DA042103. The authors declare no competing interests.
